# Supplementary material for: Complexome profiling of the Chlamydomonas psb28 mutant reveals TEF5 as an early PSII assembly factor
Source: Plant Cell. 2025 Mar 17;37(6):koaf055. doi: 10.1093/plcell/koaf055 (PMC12164590; doi:10.1093/plcell/koaf055)
Supplement: koaf055_Supplementary_Data [file koaf055_supplementary_data.zip › psb28_interactive.zip!/plots/intensity/qualityPlots/peptides/CPLD64_Cre12.g485850.t1.2.html]

### Cre12.g485850.t1.2

Trivial name: CPLD64  
SeqCoverage [median]: 34.40  
Mass: 19.68 kDa  
  
 

| Sequence | MissedCleavage | Mass [Da] | wtIntensity | muIntensity | Score |
| --- | --- | --- | --- | --- | --- |
| ASPATEEPK | 0 | 928.45 | 3159.64 | 4751.82 | 74.92 |
| FGLEASDTSEK | 0 | 1182.54 | 0.00 | 1503.99 | 116.52 |
| FPPTVALTPVVAK | 0 | 1338.79 | 0.00 | 3653.97 | 104.52 |
| SEAELATEK | 0 | 976.47 | 701.00 | 0.00 | 76.17 |
| TANKSEAELATEK | 1 | 1390.69 | 3420.62 | 6557.90 | 156.20 |
| VGTFALAYAAHK | 0 | 1247.67 | 0.00 | 4526.17 | 70.49 |
|  |  |  | 7281.3 | 20993.9 |  |
